# Comparison of Healthcare Encounters and Drug Persistence in Patients With Pulmonary Arterial Hypertension Receiving Oral Selexipag, Inhaled Iloprost, or Parenteral Treprostinil: A Retrospective Database Analysis

**DOI:** 10.36469/001c.35246

**Published:** 2022-06-08

**Authors:** Ci Song, Peter Kunovszki, Amélie Beaudet

**Affiliations:** 1 Janssen Global Commercial Strategy Organization; 2 Actelion Pharmaceuticals Ltd.

**Keywords:** pulmonary arterial hypertension, selexipag, iloprost, treprostinil, hospitalization, healthcare resource utilization, retrospective database analysis

## Abstract

**Background:** Agents targeting the prostacyclin (PGI_2_) pathway are important in managing pulmonary arterial hypertension (PAH). No head-to-head clinical trials have compared outcomes between the 3 different PGI_2_-pathway drugs most commonly available in countries with advanced healthcare: oral selexipag, inhaled iloprost, and parenteral (subcutaneous or intravenous) treprostinil.

**Objectives:** To conduct retrospective database analyses to describe characteristics of patients with PAH initiating therapy with these agents and compare the rate and risk of healthcare facility encounters and drug persistence.

**Methods:** Data were obtained from the Optum™ Clinformatics® Data Mart and Truven™ Health Analytics® MarketScan® Commercial Claims and Encounters databases from July 1, 2008, to September 30, 2020 (Optum™), or October 31, 2020 (Truven™). Patients were categorized into index-drug cohorts based on first pharmacy claims for selexipag, inhaled iloprost, or parenteral treprostinil. Eligible patients were ≥18 years of age with ≥1 ICD-9-CM or ICD-10-CM diagnosis code indicating pulmonary hypertension and no diagnosis code suggesting Group 3–5 pulmonary hypertension. Rates of hospitalization (inpatient admissions), emergency room visits, or outpatient visits per person-year were calculated. Drug persistence was measured as time to discontinuation of index drug. Multivariable analyses were performed to compare outcomes with selexipag vs inhaled iloprost and parenteral treprostinil, adjusting for baseline characteristics using inverse probability of treatment weighting.

**Results:** Overall, 583 patients were included in the Optum™ sample and 482 in the Truven™ sample. Mean (SD) age was 61.7 (14.5) and 49.3 (11.3) years, respectively; 74.4% and 75.7% of patients, respectively, were women. In the pooled samples, after adjustment for baseline characteristics, selexipag had a lower risk than inhaled iloprost or parenteral treprostinil for hospitalization (relative rate ratio [95% CI], 0.40 [0.22, 0.75], and 0.26 [0.17, 0.39]) and outpatient visits (0.66 [0.56, 0.78] and 0.76 [0.66, 0.88]). Trends toward lower risk of emergency room visits did not attain statistical significance. Drug discontinuation risk was 16% and 36% lower with selexipag vs parenteral treprostinil and inhaled iloprost, respectively.

**Conclusions:** In real-world use, selexipag appears to be associated with lower rates of hospitalization and outpatient visits than inhaled iloprost or parenteral treprostinil. Further research is required to identify factors underlying these differences.

## BACKGROUND

Pulmonary arterial hypertension (PAH) is a rare and debilitating chronic disease of the pulmonary vasculature, characterized by vascular proliferation and remodeling of the small pulmonary arteries.^1,2^ These pathological changes result in a rise in pulmonary vascular resistance and pulmonary arterial pressure, causing the nonspecific symptoms typically observed at initial presentation, including breathlessness during exercise, fatigue, chest pain, syncope, and lower extremity edema.^2–4^ Disease progression leads to worsening symptoms, frequent hospitalizations, and ultimately right-sided heart failure and premature death.^2,3,5^

Several PAH-specific drugs are currently available in Europe and North America, each of which targets abnormalities in 1 of the 3 key pathways involved in the pathophysiology of PAH for which medication is available: the prostacyclin (PGI_2_), nitric oxide, or endothelin pathways.^6^ PAH-specific therapies targeting the PGI_2_ pathway include the synthetic prostacyclin epoprostenol, the prostacyclin analogues iloprost and treprostinil, and the non-prostanoid prostaglandin I_2_ receptor agonist selexipag. All approved therapies for PAH acting on the endothelin pathway are endothelin receptor antagonists (ERAs) (bosentan, ambrisentan, and macitentan), while the nitric oxide pathway is targeted by phosphodiesterase type 5 inhibitors (PDE5is) (sildenafil and tadalafil) or the soluble guanylate cyclase stimulator (sGCS) riociguat.^6^

Current PAH treatment guidelines recommend that treatment decisions, including selection of PAH-specific agents, take into account a combination of clinical and functional variables associated with a patient’s risk for clinical worsening and death.^4,7,8^ Oral ERAs and PDE5is are currently recommended for initial therapy in low- or intermediate-risk patients, generally in initial combination therapy with a drug from each of these 2 classes, with a residual role for monotherapy in specified subtypes.^7,8^ In patients with an inadequate clinical response to monotherapy or dual therapy with an ERA and a nitric oxide pathway agent, an additional drug targeting the PGI_2_ pathway is recommended.^7,8^ Other than intravenous epoprostenol, which is usually reserved for patients with the most severe disease, parenteral treprostinil, inhaled iloprost, and oral selexipag are the drugs targeting the PGI_2_ pathway commonly available outside the United States.

In the absence of head-to-head trials comparing selexipag with other drugs targeting the PGI_2_ pathway, real-world evidence can help to fill the knowledge gap of how patient outcomes may differ between these agents. However, outcomes have not previously been compared with selexipag vs parenteral treprostinil and inhaled iloprost, the most commonly available PGI_2_-pathway comparators in countries with advanced healthcare systems.

The objective of the present retrospective database study was to compare selexipag vs inhaled iloprost and parenteral treprostinil in terms of healthcare facility encounters and drug persistence. While our preference would have been to perform these analyses using European sources, sufficient data from European countries were not readily available, given the rarity of PAH and the relatively infrequent use of inhaled iloprost and parenteral treprostinil. Following feasibility assessments, we determined that 2 large US claims databases would provide adequate data for the present analyses.

## METHODS

### Data Sources

Data for this study were obtained from the 2 healthcare claims databases: the Optum™ Clinformatics® Data Mart and Truven™ Health Analytics MarketScan^®^ Commercial Claims and Encounters (CCAE) databases. Both databases record longitudinal inpatient and outpatient medical and outpatient pharmaceutical administrative claims for individuals enrolled in health plans in the United States. The Optum™ database comprises records for over 80 million patients, most of whom are fully insured in commercial plans, though there are also some Medicare Advantage enrollees. The Truven™ CCAE database includes approximately 150 million patients enrolled in employer-sponsored health insurance plans.

In a preliminary assessment, we also explored the feasibility of using the Truven™ Medicare Supplemental and Coordination of Benefits Database, which covers health services of retirees in the United States with primary or Medicare supplemental coverage through privately insured fee-for-service, point-of-service, or capitated health plans. However, after application of our inclusion and exclusion criteria, this database yielded only 68 patients eligible for the current study and thus would have provided too few additional patients to be a meaningful complement to the Optum™ and Truven™ CCAE databases. (For simplicity, the Truven™ CCAE database is henceforth referred to as the Truven™ database.)

Drugs received by patients were identified on the basis of dispensing claims coded with National Drug Codes (NDC) or from medication-related procedures coded with Healthcare Common Procedure Coding System (HCPCS) codes. Disease diagnoses were identified from *International Classification of Diseases, Ninth or Tenth Revision, Clinical Modification* (ICD-9-CM or ICD-10-CM) codes. Medical procedures were identified using HCPCS codes.

All data used in these analyses have been anonymized and are fully compliant with the Health Insurance Portability and Accountability Act (HIPAA) privacy rules; thus, institutional review board review and approval were not required.

### Study Design

**Figure 1.**
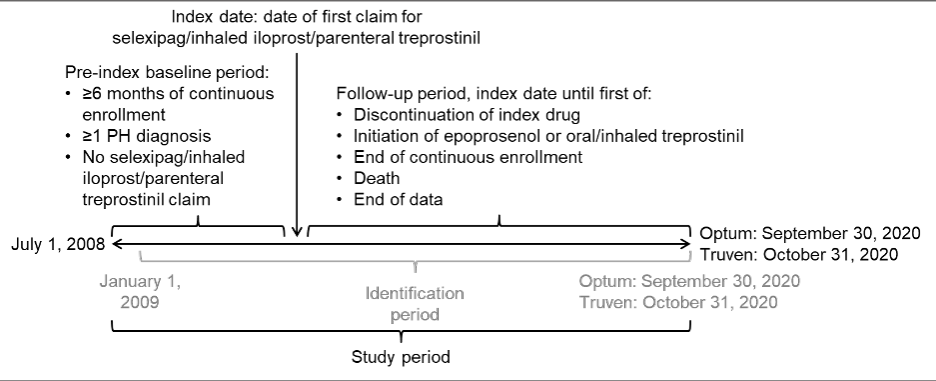
Study Design Abbreviation: PH, pulmonary hypertension.

Data were retrieved for the study period commencing on July 1, 2008, in both databases and ending on September 30, 2020, for the Optum™ database and October 31, 2020, for the Truven™ database (Figure 1). Within this study period, each patient’s index date was defined as the first date of a claim for selexipag, inhaled iloprost, or parenteral treprostinil (defined as their index drug) within the identification period of January 1, 2009, to either September 30, 2020 (Optum™), or October 31, 2020 (Truven™). The start date of the identification period allowed for a baseline period of at least 6 months before the index date to capture patients’ clinical characteristics, including pulmonary hypertension (PH) diagnoses, comorbidities, and prior medication use; the same duration of baseline period was used by McConnell et al^9^ and Dean et al^10^ in their published retrospective database analyses of selexipag vs oral treprostinil. The follow-up period for each patient was from their index date until the earliest of discontinuation of index drug, initiation of oral or inhaled treprostinil (formulations of treprostinil available only in the United States^11^) or epoprostenol, health plan disenrollment, death, or the end of the study period.

### Sample Selection

For these analyses, the focus was on new users of the drugs of interest. Accordingly, patients were excluded if they had any claim for treatments targeting the PGI_2_ pathway within the baseline period, including selexipag, iloprost, and treprostinil. Adult patients at least 18 years of age at the index date were eligible if they had at least 6 months of continuous health plan enrollment prior to the index date. Because these claims databases do not include linked medical charts that would have permitted confirmation of diagnoses, patients with PAH were ascertained using an algorithm. In addition to at least 1 pharmacy claim for selexipag, inhaled iloprost, or parenteral treprostinil at the index date, inclusion required at least 1 medical claim in the baseline period or on the index date with an ICD-9-CM or ICD-10-CM diagnostic code for PH, namely, ICD-9-CM 416.0 (primary pulmonary hypertension), 416.8 (other chronic pulmonary heart disease; pulmonary hypertension, secondary) or 416.9 (chronic pulmonary heart disease, unspecified), or ICD-10-CM I27.0 (primary pulmonary hypertension), I27.2 (other secondary pulmonary hypertension), I27.20 (pulmonary hypertension, unspecified), I27.21 (secondary pulmonary arterial hypertension), I27.89 (other specified pulmonary heart diseases), or I27.9 (pulmonary heart disease, unspecified).

To increase the specificity of the algorithm for patients with PAH rather than other types of PH, patients were excluded if they had any claim with a diagnosis code for PH in Groups 3-5 as defined by the 3rd and 4th World Symposium on Pulmonary Hypertension (WSPH)^12,13^ within the 6-month baseline period prior to the index date (see **Supplementary Table S1** for the diagnosis codes). However, patients with a diagnosis code for PH in Group 2 (ie, PH due to left-sided heart disease) were not excluded since a substantial proportion of patients with PAH also have comorbid left-sided heart disease.^14–16^

Baseline characteristics for each patient were retrieved during the 6-month baseline period. Demographic variables measured were age at index date (based on recorded birth year), gender, index year, insurance type, and geographic region. Background PAH medications were identified and classified as monotherapy or combination therapy based on whether only 1 or multiple different PAH-specific agents, respectively, were prescribed during the baseline period. The Charlson Comorbidity Index (CCI) score was calculated using the methods of Charlson et al^17,18^ and Deyo et al.^19^ Selected clinical comorbidities of interest were categorized as PAH-related, including connective tissue disease, congenital heart disease (CHD), and portal hypertension, or other comorbidities of interest, including depression, hypertension, diabetes, chronic obstructive pulmonary disease (COPD), asthma, digital ulcers, heart failure, hyperlipidemia, and obesity. Selected comorbidities were defined based on medical claims using ICD-9-CM and ICD-10-CM codes (see **Supplementary Table S1**).

### Treatment Cohort Assignment

Each patient was assigned to 1 of 3 treatment cohorts—selexipag, inhaled iloprost, or parenteral treprostinil—based on their index drug. The selexipag cohort was the comparator for pairwise statistical comparisons, with the inhaled iloprost and parenteral treprostinil cohorts as reference groups.

Continuous treatment with the index drug was assessed based on the number of consecutive days with drug supply. A gap between consecutive prescriptions was considered to represent a break in a continuous treatment episode if the gap exceeded 90 days after the end of the days of supply from the last prescription. If the number of days of supply was not present in the prescription record, a 30-day supply was imputed based on an exploratory analysis that showed that the median prescription interval for all 3 of these drugs was approximately 30 days.

### Sample Weighting

Especially given their different routes of administration, it cannot be assumed that patients are randomly assigned to the 3 treatment cohorts (ie, the PGI_2_-pathway drugs in this study are unlikely to have been prescribed with equal probability to patients without regard to differences in their characteristics) (see prescribing criteria in **Supplementary Table S2**). To reduce potential bias due to confounding by indication in the different index-drug cohorts, we adjusted for multiple baseline characteristics listed above using the inverse probability of treatment weighting (IPTW) method as described by Curtis et al.^20^ IPTW and propensity score (PS)–matching are the 2 main methods recommended for adjusting for potential confounders in studies comparing effectiveness and safety in different treatment groups using secondary data.^21^ Although the 2 methods generally perform similarly, a simulation study has shown that IPTW estimations are systematically less biased and mean standard errors are smaller than those reached by PS-matching when the sample size is small.^21^ Considering the sample size is not large in our study, we selected the IPTW method.

A logistic regression model was used to calculate the PS for each patient in the treatment cohorts, which reflects the predicted probability of receiving selexipag or the comparator treatment, conditional on observed baseline characteristics. The IPTW was then calculated as the inverse of the PS. A matching weight based on R package *psw.mw* was applied to achieve stabilized computation.^22^ Cohort balance was checked for each variable and considered to be well balanced after weighting, as the standardized mean difference between 2 treatment cohorts was less than 10% for each variable. If one baseline variable were to be imbalanced after weighting, it would be further included as a covariate in the multivariable models.

Multiple demographic characteristics and clinical characteristics were incorporated in the IPTW. Demographic characteristics included age, sex, insurance type, and residence region. Clinical characteristics included other PAH treatments (ie, ERAs and/or PDE-5is), CCI score, as well as a series of comorbidities at baseline (ie, CHD, CTD, portal hypertension, hypertension, obesity, hyperlipidemia, heart failure, COPD, diabetes, and depression). Asthma and digital ulcer at baseline were not included in the IPTW because no patient had either asthma or digital ulcer at baseline among selexipag users.

### Outcomes

Outcomes assessed were healthcare facility encounters and drug persistence. Healthcare facility encounters were estimated as rates of hospitalization (ie, inpatient admissions), emergency room (ER) visits, or outpatient visits per person-year. Drug persistence was measured as risk of discontinuation (ie, time to discontinuation of the index drug), based on the definition of the first treatment episode as described above.

### Statistical Analysis

Descriptive statistics for baseline characteristics are reported as counts and percentages for categorical variables, and mean and SD for continuous variables.

Multivariable analyses were performed to compare outcomes with selexipag vs inhaled iloprost and parenteral treprostinil, after being weighted by IPTW. Based on goodness-of-fit, an appropriate model was chosen between weighted negative binominal regression and weighted Poisson regression with a logarithm link function and offsetting the logarithm of follow-up time for estimating the relative rate ratios with 95% CI of healthcare facility encounters. Weighted Cox proportional hazards regression models were used to estimate hazard ratios with 95% CIs for index-drug persistence. Multivariable analyses were performed within each database separately and on data pooled from both databases. Furthermore, a meta-analysis was conducted to derive the pooled estimate of Optum™ and Truven™ using the inverse-variance fixed-effect model. Statistical analyses were performed using R 4.0.2 (R Foundation for Statistical Computing, Vienna, Austria).

## RESULTS

### Patient Characteristics

**Figure 2.**
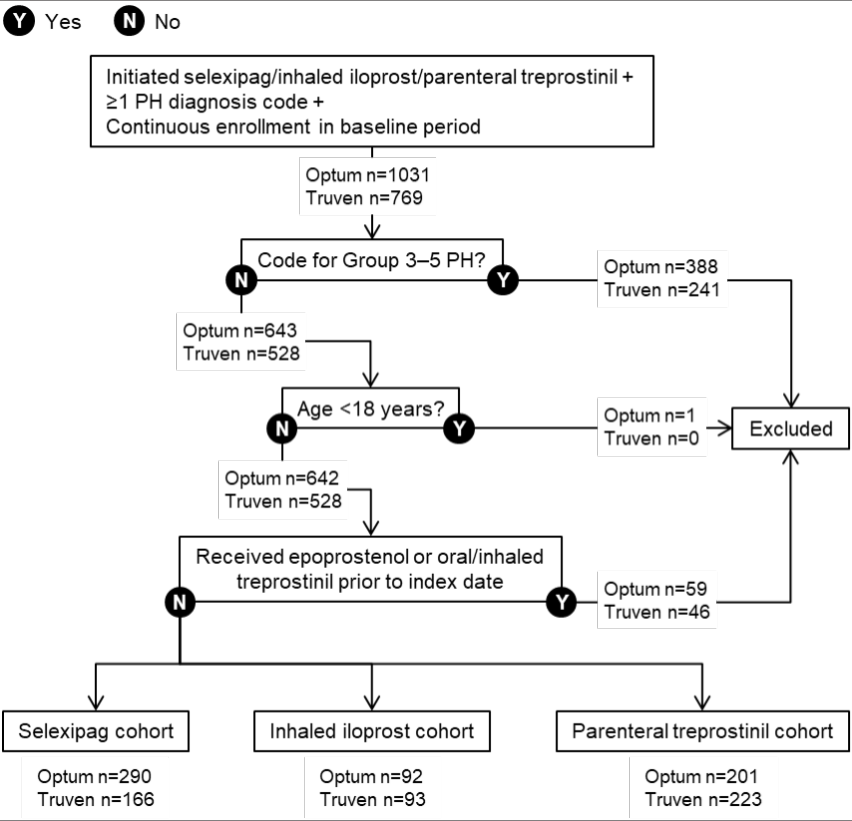
Ascertainment of Patients With Pulmonary Arterial Hypertension Abbreviation: PH, pulmonary hypertension.

After the application of all eligibility criteria, 583 patients were included in the overall Optum™ population and 482 in the Truven™ population (Figure 2). The majority of patients were women (74.4% and 75.7% for Optum™ and Truven™, respectively; Table 1). Mean (SD) age was 61.7 (14.5) and 49.3 (11.3) years in the Optum™ and Truven™ samples, respectively. In the Optum™ cohort, 51.5% of patients had Medicare coverage and the remainder were in commercial plans; all patients in the Truven™ sample had commercial coverage.

**Table 1.** Baseline Characteristics and Index Year

**Optum™**						**Truven™**		
	**Selexipag (n=290)**	**Inhaled Iloprost (n=92)**	**Parenteral Treprostinil (n=201)**	**Total (n=583)**	**Selexipag (n=166)**	**Inhaled Iloprost (n=93)**	**Parenteral Treprostinil (n=223)**	**Total (n=482)**
Age, years, mean (SD)	63.8 (14.2)	63.3 (13.8)	57.9 (14.6)	61.72 (14.5)	50.1 (11.5)	51.7 (9.8)	47.7 (11.5)	49.3 (11.3)
Female, n (%)	213 (73.4)	66 (71.7)	155 (77.1)	434 (74.4)	131 (78.9)	68 (73.1)	166 (74.4)	365 (75.7)
Insurance type, n (%)								
Commercial	181 (62.4)	39 (42.4)	80 (39.8)	300 (51.5)	166 (100.0)	93 (100.0)	223 (100.0)	482 (100.0)
Medicare	109 (37.6)	53 (57.6)	121 (60.2)	283 (48.5)	0	0	0	0
US geographic region, n (%)								
East North Central	29 (10.0)	8 (8.7)	49 (24.4)	86 (14.8)	24 (14.5)	12 (12.9)	45 (20.2)	81 (16.8)
East South Central	15 (5.2)	2 (2.2)	3 (1.5)	20 (3.4)	6 (3.6)	7 (7.5)	14 (6.3)	27 (5.6)
Middle Atlantic	22 (7.6)	4 (4.3)	10 (5.0)	36 (6.2)	15 (9.0)	14 (15.1)	19 (8.5)	48 (10.0)
Mountain	32 (11.0)	11 (12.0)	33 (16.4)	76 (13.0)	7 (4.2)	3 (3.2)	20 (9.0)	30 (6.2)
New England	9 (3.1)	4 (4.3)	8 (4.0)	21 (3.6)	4 (2.4)	6 (6.5)	7 (3.1)	17 (3.5)
Pacific	25 (8.6)	19 (20.7)	27 (13.4)	71 (12.2)	22 (13.3)	11 (11.8)	23 (10.3)	56 (11.6)
South Atlantic	99 (34.1)	24 (26.1)	34 (16.9)	157 (26.9)	36 (21.7)	20 (21.5)	44 (19.7)	100 (20.7)
West North Central	18 (6.2)	10 (10.9)	13 (6.5)	41 (7.0)	5 (3.0)	3 (3.2)	10 (4.5)	18 (3.7)
West South Central	40 (13.8)	10 (10.9)	24 (11.9)	74 (12.7)	22 (13.3)	15 (16.1)	33 (14.8)	70 (14.5)
Unknown	1 (0.3)	0 (0.0)	0 (0.0)	1 (0.2)	25 (15.1)	2 (2.2)	8 (3.6)	35 (7.3)
CCI score, mean (SD)	4.1 (3.2)	3.8761 (2.8)	4.0 (2.8)	4.0 (3.0)	2.6 (2.5)	2.4(2.1)	2.9 (2.4)	2.7 (2.4)
Comorbidities, n (%)								
CHD	27 (9.3)	9 (9.8)	22 (10.9)	58 (9.9)	22 (13.3)	13 (14.0)	26 (11.7)	61 (12.7)
CTD	49 (16.9)	20 (21.7)	47 (23.4)	116 (19.9)	22 (13.3)	21 (22.6)	52 (23.3)	95 (19.7)
Portal hypertension	16 (5.5)	6 (6.5)	22 (10.9)	44 (7.5)	13 (7.8)	2 (2.2)	20 (9.0)	35 (7.3)
Hypertension	228 (78.6)	70 (76.1)	143 (71.1)	441 (75.6)	93 (56.0)	53 (57.0)	125 (56.1)	271 (56.2)
Obesity	84 (29.0)	21 (22.8)	58 (28.9)	163 (28.0)	47 (28.3)	10 (10.8)	44 (19.7)	101 (21.0)
Hyperlipidemia	156 (53.8)	34 (37.0)	87 (43.3)	277 (47.5)	48 (28.9)	27 (29.0)	51 (22.9)	126 (26.1)
Heart failure	166 (57.2)	49 (53.3)	138 (68.7)	353 (60.5)	60 (36.1)	45 (48.4)	134 (60.1)	239 (49.6)
Digital ulcer	0 (0.0)	1 (1.1)	1 (0.5)	2 (0.3)	0 (0.0)	1 (1.1)	6 (2.7)	7 (1.5)
Asthma	0 (0.0)	22 (23.9)	23 (11.4)	45 (7.7)	0 (0.0)	12 (12.9)	28 (12.6)	40 (8.3)
COPD	127 (43.8)	43 (46.7)	87 (43.3)	257 (44.1)	38 (22.9)	26 (28.0)	49 (22.0)	113 (23.4)
Diabetes	110 (37.9)	41 (44.6)	58 (28.9)	209 (35.8)	44 (26.5)	23 (24.7)	50 (22.4)	117 (24.3)
Depression	45 (15.5)	11 (12.0)	44 (21.9)	100 (17.2)	17 (10.2)	9 (9.7)	36 (16.1)	62 (12.9)
Baseline PAH-specific treatment, n (%)								
ERA + PDE5i combination	167 (57.6)	30 (32.6)	51 (25.4)	248 (42.5)	98 (59.0)	42 (45.2)	46 (20.6)	186 (38.6)
ERA monotherapy	28 (9.7)	31 (33.7)	27 (13.4)	86 (14.8)	21 (12.7)	26 (28.0)	27 (12.1)	74 (15.4)
PDE5i/sGCS monotherapy	54 (18.6)	14 (15.2)	46 (22.9)	114 (19.6)	32 (19.3)	17 (18.3)	60 (26.9)	109 (22.6)
None	41 (14.1)	17 (18.5)	77 (38.3)	135 (23.2)	15 (9.0)	8 (8.6)	90 (40.4)	113 (23.4)
Index year, n (%)								
2009	0 (0.0)	23 (25.0)	21 (10.4)	44 (7.5)	0 (0.0)	25 (26.9)	25 (11.2)	50 (10.4)
2010	0 (0.0)	14 (15.2)	18 (9.0)	32 (5.5)	0 (0.0)	23 (24.7)	25 (11.2)	48 (10.0)
2011	0 (0.0)	24 (26.1)	18 (9.0)	42 (7.2)	0 (0.0)	11 (11.8)	24 (10.8)	35 (7.3)
2012	0 (0.0)	5 (5.4)	16 (8.0)	21 (3.6)	0 (0.0)	12 (12.9)	39 (17.5)	51 (10.6)
2013	0 (0.0)	13 (14.1)	17 (8.5)	30 (5.1)	0 (0.0)	8 (8.6)	18 (8.1)	26 (5.4)
2014	0 (0.0)	4 (4.3)	13 (6.5)	17 (2.9)	0 (0.0)	8 (8.6)	25 (11.2)	33 (6.8)
2015	0 (0.0)	5 (5.4)	15 (7.5)	20 (3.4)	0 (0.0)	4 (4.3)	18 (8.1)	22 (4.6)
2016	50 (17.2)	3 (3.3)	16 (8.0)	69 (11.8)	52 (31.3)	1 (1.1)	12 (5.4)	65 (13.5)
2017	51 (17.6)	1 (1.1)	24 (11.9)	76 (13.0)	28 (16.9)	1 (1.1)	5 (2.2)	34 (7.1)
2018	63 (21.7)	0 (0.0)	17 (8.5)	80 (13.7)	29 (17.5)	0 (0.0)	15 (6.7)	44 (9.1)
2019	68 (23.4)	0 (0.0)	20 (10.0)	88 (15.1)	35 (21.1)	0 (0.0)	10 (4.5)	45 (9.3)
2020	58 (20.0)	0 (0.0)	6 (3.0)	64 (11.0)	22 (13.3)	0 (0.0)	7 (3.1)	29 (6.0)

Abbreviations: CCI, Charlson Comorbidity Index; CHD, congenital heart disease; COPD, chronic obstructive pulmonary disease; CTD, connective tissue disease; ERA, endothelin receptor antagonists; PAH, pulmonary arterial hypertension; PDE5i, phosphodiesterase type 5 inhibitor; PH, pulmonary hypertension; sGCS, soluble guanylate cyclase stimulator.

Hypertension, hyperlipidemia, heart failure, COPD, and diabetes were the most common comorbidities, and there was no clear trend for the prevalence of comorbidities to differ by index drug. The prevalence of comorbidities was higher in the Optum™ sample than in the Truven™ sample, for the CCI and for individual comorbidities (Table 1). This difference was expected given the inclusion of older patients on Medicare in the Optum™ database.

In all treatment cohorts, the majority of patients used an ERA and/or a PDE5i as background therapy, but the proportion on background therapy was highest in the selexipag cohort and lowest in the parenteral treprostinil cohort (Table 1). Use of background combination therapy (ie, dual therapy with an ERA plus a PDE5i/sGCS) was more than twice as frequent for patients on selexipag compared with parenteral treprostinil, and substantially higher compared with inhaled iloprost.

### Index Year

Cohort distributions by index year are reported in Table 1. No patients had selexipag claims prior to 2016, reflecting the US approval of this drug in late December 2015. There were declining prescriptions over time for inhaled iloprost, with no claims after 2017. In contrast, there was no clear trend for change over time in prescriptions of parenteral treprostinil.

### Follow-up

Mean (SD) follow-up time was 359.2 (438.0) days in the Optum™ sample and 362.2 (440.8) days in the Truven™ sample (Table 2). Mean follow-up duration was longer in the parenteral treprostinil cohort than in the inhaled iloprost cohort in both databases (405.3 vs 284.8 in the Optum™ sample and 383.7 vs 324.6 in the Truven™ sample).

**Table 2.** Follow-up Duration and Rate of Healthcare Facility Encounters in the Optum™ and Truven™ Samples

	**Optum™**				**Truven™**			
	**Selexipag (n=290)**	**Inhaled Iloprost (n=92)**	**Parenteral Treprostinil (n=201)**	**Total (n=583)**	**Selexipag (n=166)**	**Inhaled Iloprost (n=93)**	**Parenteral Treprostinil (n=223)**	**Total (n=482)**
Follow-up duration, months	350.7 (366.6)	284.8 (440.1)	405.3 (520.8)	359.2 (438.0)	354.4 (373.5)	324.6 (501.6)	383.7 (460.3)	362.2 (440.8)
Rate per person-year								
Inpatient admissions	0.3 (1.7)	1.3 (3.6)	1.0 (2.3)	0.7 (2.3)	0.5 (1.7)	0.6 (1.7)	2.3 (4.7)	1.3 (3.5)
Outpatient visits	21.3 (20.1)	29.7 (28.0)	21.4 (17.1)	22.7 (20.8)	39.6 (42.7)	52.9 (36.3)	63.6 (48.4)	53.2 (45.5)
ER visits	0.5 (2.0)	0.5 (2.5)	0.3 (1.3)	0.4 (1.9)	1.3 (3.6)	0.9 (2.2)	2.0 (3.3)	1.6 (3.3)

Data are reported as mean (SD).

Abbreviation: ER, emergency room.

### Healthcare Facility Encounters

Outpatient visits were the most frequently occurring healthcare facility encounters, followed by ER visits and hospitalizations (Table 2).

**Figure 3.**
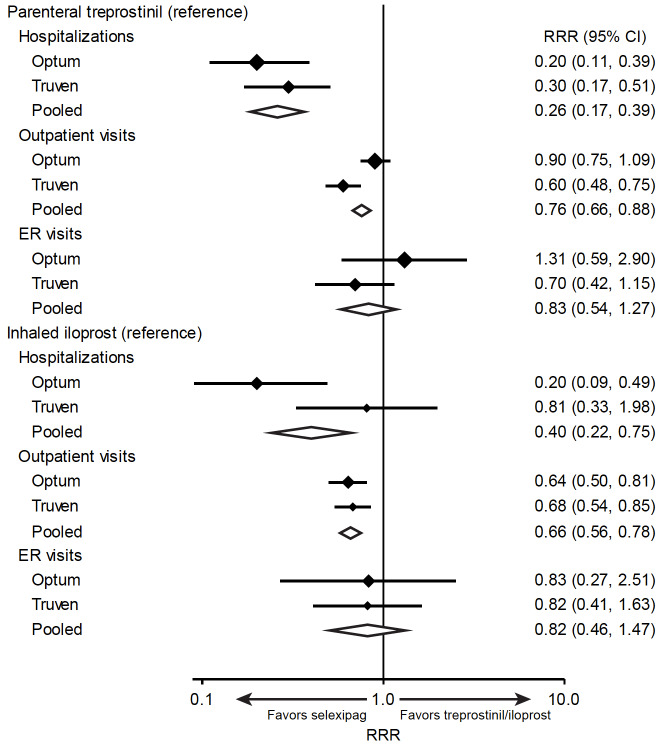
Relative Rate Ratios for Hospitalizations (Inpatient Admissions), Outpatient Visits, and Emergency Room Visits for Selexipag vs Inhaled Iloprost and Parenteral Treprostinil For Optum™ and Truven™ results, solid diamonds and bars indicate relative rate ratio and 95% CI, respectively; diamond size is proportional to sample size. For pooled results, the centers and widths of open diamonds indicate relative rate ratio and 95% CI, respectively. Relative rate ratios were estimated with either negative binominal regression or weighted Poisson regression based on goodness-of-fit. Abbreviations: ER, emergency room; RRR, relative rate ratio.

After adjustment for baseline characteristics, selexipag had significantly lower rates of hospitalization and outpatient visits than either inhaled iloprost or parenteral treprostinil in the pooled estimate (Figure 3). The same trend was present in both the Optum™ and Truven™ samples.

There was a trend toward a lower rate of ER visits for selexipag compared with inhaled iloprost, but this did not attain statistical significance in either database. Selexipag had a trend toward a lower rate of ER visits compared with parenteral treprostinil in the Truven™ database but not in the Optum™ database (Figure 3).

### Drug Persistence

**Figure 4.**
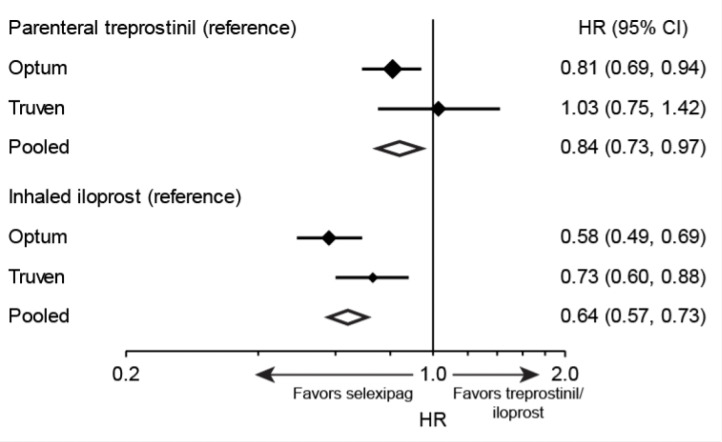
Hazard Ratios for Drug Discontinuation With Selexipag vs Inhaled Iloprost and Parenteral Treprostinil For Optum™ and Truven™ results, solid diamonds and bars indicate hazard ratio and 95% CI, respectively; diamond size is proportional to sample size. For pooled results, the centers and widths of open diamonds indicate hazard ratio and 95% CI, respectively. Hazard ratios were estimated with weighted Cox proportional hazards regression models. Abbreviation: HR, hazard ratio.

Overall, higher drug persistence with selexipag was observed compared to both inhaled iloprost and parenteral treprostinil (Figure 4). The risk of drug discontinuation of selexipag was 16% lower compared with parenteral treprostinil and 36% lower compared with inhaled iloprost. Trends were similar in the Optum™ and Truven™ samples for the comparison of selexipag with inhaled iloprost, but a significantly lower rate of discontinuation with selexipag compared with parenteral treprostinil was observed only in the Optum™ sample.

## DISCUSSION

This study provides real-world evidence on the characteristics and outcomes of patients receiving 3 PAH-specific therapies all targeting the PGI_2_ pathway but administered by 3 different routes: oral, inhaled, and parenteral. After adjusting for several potentially confounding variables, selexipag was found to be associated with significantly lower rates of hospitalizations and outpatient visits than either parenteral treprostinil or inhaled iloprost. Lower hospitalization and outpatient attendance may reflect real differences in the relative ability of these agents to delay disease worsening and thus reduce these measures of morbidity, but the possibility that the observed results may have been influenced by unmeasured confounders precludes a firm conclusion.

Improved drug persistence may be expected for an agent that has a less burdensome route of administration and a more favorable side-effect profile.^23,24^ Pooled point estimates suggested significantly better drug persistence for oral selexipag than for parenteral treprostinil and inhaled iloprost.

Four drugs targeting the PGI_2_ pathway are approved for treating PAH in most countries with advanced healthcare systems: epoprostenol, treprostinil, iloprost, and selexipag.^25^ Real-world evidence is particularly valuable when comparing these agents because the quality of clinical trial evidence supporting each of these drugs differs to an extent that makes comparison challenging. Epoprostenol was the first PAH-specific therapy to be approved (in 1995) and remains the gold standard for high-risk patients,^26^ with the highest recommendation in clinical practice guidelines,^7,8^ on the basis of improved survival vs placebo in the 12-week Primary Pulmonary Hypertension Study.^27^ Epoprostenol is not available in all countries, including some European nations such as Finland and Slovakia,^28^ and may not be widely used even where it has marketing authorization, as is the case in Germany.^29^

Treprostinil is a prostacyclin analogue with a longer elimination half-life than epoprostenol.^30^ Although parenteral, inhaled, and oral formulations are available in the United States,^11^ treprostinil is approved only for continuous subcutaneous or intravenous administration in Europe and most of the world. Treprostinil was approved in Europe in 2005,^31^ based on improvement in exercise capacity (specifically, 6-minute walk distance; 6MWD), symptoms, and hemodynamics vs placebo in a 12-week RCT of subcutaneous infusion.^32^ Continuous intravenous treprostinil was approved on the basis of bioequivalence between the intravenous and subcutaneous routes of administration but is recommended only for patients unable to tolerate subcutaneous administration.^7,30^ The 12-week TRUST-1 RCT of intravenous treprostinil was terminated early due to safety concerns,^33^ and international clinical practice guidelines note that the resulting data are considered unreliable.^7^

The prostacyclin analogue iloprost was approved for inhaled administration in Europe in 2003 on the basis of the 12-week AIR study, which demonstrated an improvement vs placebo on the composite primary endpoint of improvement in New York Heart Association functional class and 6MWD in the absence of clinical deterioration, as well as on symptoms and hemodynamics.^31,34^ Although continuous intravenous iloprost has been used off-label for patients with PAH in Germany and some other European countries,^35^ the intravenous route of administration for iloprost is currently approved for PAH treatment only in New Zealand.^11^

Oral selexipag was approved in the United States in 2015 and in Europe in 2016,^36^ on the basis of the event-driven GRIPHON RCT, in which selexipag significantly reduced the risk of morbidity and mortality events compared with placebo.^37^ In contrast to the short-term (12-week) pivotal trials for subcutaneous treprostinil and inhaled iloprost,^32,34^ which were conducted in an era when PAH drugs were approved primarily on the basis of improvement in exercise capacity and symptoms, GRIPHON was a long-term (median follow-up to end of treatment of 70.7 weeks for selexipag and 63.7 weeks for placebo) outcomes study that employed a composite primary endpoint measuring morbidity and mortality,^37^ as recommended at the 4th and 5th WSPH.^38,39^

Agents targeting the PGI_2_ pathway have characteristic adverse events (AEs) associated with their mechanism of action, including headache, diarrhea, flu-like symptoms, jaw pain, muscle spasm, flushing, and nausea.^11^ These agents also have major differences in their side-effect profiles and burden of dosing related to their different routes of administration. Agents that are taken by parenteral or inhaled routes are associated with substantial administration-related unwanted events, notably infusion site pain and reaction for subcutaneous infusion, catheter occlusion and line infection for intravenous infusion, and cough for inhaled therapies.^11^ Continuous parenteral infusion also requires complex and time-consuming aseptic procedures for drug preparation and administration, and care of the portable infusion pump.^11,26,40^ Inhaled iloprost requires 6 to 9 doses daily due to its short half-life,^34^ and each dosing takes up to 15 minutes.^41,42^ Device setup, cleaning, and packing require additional time.^24^ As a result of their side-effect profile and administration burden, these agents are typically reserved for high-risk patients.

Oral therapy provides greater convenience and could enable use of these agents earlier in the disease course.^43^ A recent analysis by McConnell et al^9^ of data from the Optum Clinformatics Data Mart healthcare claims database in the US indicated that selexipag is associated with a significantly lower risk and rate of PH-related and all-cause hospitalization than oral treprostinil, although this was not seen in an analysis by Dean et al^10^ using the Truven™ Health Analytics MarketScan® CCAE database.

While these comparative studies on two oral PGI_2_-pathway drugs are of obvious interest in the United States, comparisons between selexipag and inhaled iloprost and parenteral treprostinil are more relevant globally because these are the drugs clinicians and payers must choose between in much of the world for patients with PAH requiring a PGI_2_-pathway agent.

In our study, most patients receiving one of the drugs of interest were also using an ERA and/or a PDE-5i/sGCS (76.8% and 76.6% in the Optum™ and Truven™ samples, respectively). Historically, ERAs and PDE5is have usually been selected as first-line therapy for patients with PAH at low and moderate risk.^44^ This choice reflects clinical practice guidelines^7^ and a number of practical advantages, including oral administration, a relatively favorable safety and tolerability profile, the availability of long-term RCT data, extensive clinical experience with their use, cost considerations, and simple posology not requiring complex titration. Although drugs targeting the PGI_2_ pathway have demonstrated benefits in function, morbidity, and mortality, their side-effect profiles and routes of delivery can be more challenging, and dose titration is highly individualized, requiring more experience from clinicians and greater commitment from patients.^43,45^ Consequently, agents targeting the PGI_2_ pathway have typically been reserved as add-on therapy for patients who continue to show evidence of clinical worsening despite treatment with an ERA and/or a PDE5i/sGCS.^44^ In recent years, there has been a shift in recommended clinical practice away from initial monotherapy with either an ERA or a PDE5i/sGCS toward initial dual combination therapy with agents from both classes, even for many patients at low or intermediate risk.^8^ There is also increasing recognition of the importance of treatment escalation to triple combination therapy by adding on a drug targeting the PGI_2_ pathway for those patients who do not achieve and maintain a low risk profile on a dual ERA–PDE5i/sGCS regimen.^46^ Prior to the approval of selexipag, inhaled iloprost and parenteral treprostinil were the only drugs available in Europe to enable triple combination therapy in patients not yet at high enough risk to warrant initiation of intravenous epoprostenol (where approved), which is associated with potentially serious AEs, including catheter-related soft-tissue infection, sepsis, and thrombosis.^26,40^ The availability of oral selexipag allows triple combination therapy to be implemented earlier in the disease course, without the burden associated with the inhaled and parenteral routes of administration of the prostacyclin analogues.^43,46^

In our study, compared with patients receiving inhaled iloprost or parenteral treprostinil, those receiving selexipag had a higher prevalence of triple combination therapy with the PGI_2_-pathway agent plus both an ERA and a PDE5i/sGCS. This difference could reflect prescriber awareness of the fact that in the GRIPHON trial selexipag demonstrated significant incremental benefit on the composite primary morbidity/mortality endpoint in the subgroup of patients receiving background dual combination therapy.^37,47^

### Study Strengths and Limitations

An important strength of our study was the use of an algorithm to ascertain patients with PAH, as clinical validation of PAH is not available in the claims databases. The algorithm includes both a diagnosis code and a drug code indicative of PAH, which is likely to identify fewer false-positive patients than using only one of these two methods.^48–50^ Notably, the preponderance of women in both study cohorts (approximately three-fourths of patients in both samples) was consistent with the gender distribution reported for patients in the United States with diagnosed PAH in the REVEAL registry, of whom 79% were women.^51,52^

Our study also had limitations. As there is no unique ICD-9 or ICD-10 code that distinguishes all PAH subtypes from other forms of PH, ascertaining patients on the basis of these codes could have led to misclassification of some patients.^49,53^ Assessment of the accuracy of our algorithm would require application to different databases and direct validation against patients with PAH diagnoses directly confirmed by chart review, as has been recommended for other algorithms in this therapeutic area.^48^ However, it was not possible to perform these checks within the scope of our study. Similarly, diagnoses of other types of PH and comorbidities ascertained from codes could not be validated by review of individual patient charts.

The index year range differed for the 3 drugs of interest: whereas inhaled iloprost and parenteral treprostinil were both available throughout the study period, selexipag was not marketed in the United States until 2016. Following the introduction of selexipag, parenteral treprostinil continued to be prescribed whereas inhaled iloprost prescriptions declined to zero. Interpretation of the study results is limited by the fact that the multivariable analyses did not adjust for index year. In addition, the administrative claims databases used in this study do not contain information on clinical variables known to be prognostic for clinical worsening and death in PAH, such as functional class, 6MWD, right-sided heart catheterization findings, or levels of the biomarker N-terminal fragment of pro–brain natriuretic peptide.^52^ This limitation precludes a more detailed characterization of the risk profiles of the patients receiving the drugs of interest and prevents adjustment for these confounders in the multivariable analyses.

Another study limitation is that administrative claims do not provide information on whether the prescriptions were filled and taken as prescribed, which should be considered when interpreting our adherence and outcomes data. As this study included US patients who are commercially insured, these results may not be generalizable to patients with different coverage, to the US population as a whole, or to patients living outside the United States. Finally, like all retrospective database studies, it is not possible to draw firm conclusions regarding causality of the associations found in this study.

## CONCLUSIONS

The results of our retrospective database analysis suggest that oral selexipag is associated with lower rates of hospitalizations and outpatient visits than inhaled iloprost or parenteral treprostinil as well as a lower risk of drug discontinuation. Further research is required to identify factors that may underlie these differences.

### Author Contributions

C.S. and A.B. conceived of the study and participated in study design. C.S. and P.K. analyzed the data. All authors contributed to data interpretation, and manuscript drafting and critical revision. All authors approved the final manuscript and consented to submit it for publication. All authors had full access to all the data in this study and agree to be accountable for all aspects of the work, in ensuring that questions related to the accuracy or integrity of any part of the work are appropriately investigated and resolved.

### Ethical Considerations

This retrospective study did not involve any interventional research requiring ethics committee review.

### Conflicts of Interest

C.S. was an employee of Janssen Global Commercial Strategy Organization and held shares of Janssen when this research was conducted (she is currently a Research Scientist at Evidera/PPD, Inc). P.K. is an employee of Janssen Pharmaceutical Companies of Johnson & Johnson. A.B. is an employee of Actelion Pharmaceuticals Ltd and has stock options in Johnson & Johnson. All authors received medical writing support on this article, funded by Actelion Pharmaceuticals Ltd.

### Data Sharing

The data for these analyses were made available to the authors by third-party license from Optum Clinformatics and Truven Health Analytics, two commercial data providers in the US. Under the licensing agreements, the authors cannot share these data themselves. Other researchers could access the data by purchase from these providers, and the inclusion criteria specified in the Methods section would allow them to identify the same patient cohorts we used for these analyses. No authors had special privileges to access these data, and other researchers would be able to access the data via third-party license in the same manner as the authors.

## Supplementary Material

Online Supplementary Material

Online Supplementary Material
