# Supplementary material for: Comparison of Healthcare Encounters and Drug Persistence in Patients With Pulmonary Arterial Hypertension Receiving Oral Selexipag, Inhaled Iloprost, or Parenteral Treprostinil: A Retrospective Database Analysis
Source: J Health Econ Outcomes Res. 2022 Jun 8;9(1):151–60. doi: 10.36469/001c.35246 (PMC9178228; doi:10.36469/001c.35246)
Supplement: Online Supplementary Material [file jheor_2022_9_1_35246_91845.pdf]

Journal of Health Economics and Outcomes Research

Online Supplementary Material

Comparison of Healthcare Encounters and Drug Persistence in Patients With Pulmonary Arterial Hypertension Receiving Oral Selexipag, Inhaled Iloprost, or Parenteral Treprostinil: A Retrospective Database Analysis. *JHEOR*. 2022;9(1):151-160. doi:10.36469/jheor.2022.35246

Table S1: Codes Used to Ascertain Pulmonary Hypertension Diagnoses, Drugs, Comorbidities, and Treatment-related Unwanted Events

Table S2: Prescribing Criteria for the Drugs of Interest

This supplementary material has been provided by the authors to give readers additional information about their work.

This is an open-access article distributed under the terms of the Creative Commons Attribution 4.0 International License (CCBY-4.0). View this license's legal deed at http://creativecommons.org/licenses/by/4.0 and legal code at http://creativecommons.org/licenses/by/4.0/legalcode for more information.

Table S1. Codes Used to Ascertain Pulmonary Hypertension Diagnoses, Drugs, Comorbidities, and Treatment-related Unwanted Events

Description	Code
**Inclusion diagnoses**	
	**ICD-9-CM**
Primary pulmonary hypertension	416.0
Other chronic pulmonary heart diseases	416.8
Chronic pulmonary heart disease, unspecified	416.9
	**ICD-10-CM**
Primary pulmonary hypertension	I27.0
Other secondary pulmonary hypertension	I27.2
Pulmonary hypertension, unspecified	I27.20
Secondary pulmonary arterial hypertension	I27.21
Other specified pulmonary heart diseases	I27.89
Pulmonary heart disease, unspecified	I27.9
**Exclusion diagnoses**	
	**ICD-9-CM**
Cystic fibrosis	277.0x
Chronic and other pulmonary manifestations due to radiation	508.1
Postinflammatory pulmonary fibrosis	515
Idiopathic interstitial pneumonia	516.3x
Adult pulmonary Langerhans cell histiocytosis	516.5
Inferior vena cava filter	38.7
Pulmonary embolism and infarction	415.1
Other venous thrombosis and embolism	V12.51
Sarcoidosis	135
Glycogenosis	271.0
Lipidoses	272.7
Acquired hemolytic anemias	283
Autoimmune hemolytic anemias	283.0
Non-autoimmune hemolytic anemias	283.1x
Hemoglobinuria due to hemolysis from external causes	283.2
Acquired hemolytic anemia, unspecified	283.9
Lymphangioleiomyomatosis	516.4
	**ICD-10-CM**
Pulmonary hypertension due to lung diseases and hypoxia	I27.23
Cystic fibrosis	E84.x
Other interstitial pulmonary diseases	J84.x
Pulmonary thromboembolism	I26
Acquired hemolytic anemia	D59.x
Sarcoidosis	D86.x
Glycogen storage disease	E74.0x
Gaucher disease	E75.22
	**CPT-4**
Inferior vena cava filter	36010
Inferior vena cava filter	37620
Inferior vena cava filter	75825
Inferior vena cava filter	75940

Table S1. Codes Used to Ascertain Pulmonary Hypertension Diagnoses, Drugs, Comorbidities, and Treatment-related Unwanted Events

**Description**	**Code**
	**HCPCS**
Vena cava filter	C1880
**Drugs**	
Selexipag	**NDC**
Uptravi®	66215060206
Uptravi®	66215060214
Uptravi®	66215060406
Uptravi®	66215060606
Uptravi®	66215060806
Uptravi®	66215061006
Uptravi®	66215061206
Uptravi®	66215061406
Uptravi®	66215061606
Uptravi® Titration Pack	66215062820
Epoprostenol	
Flolan®	00173051700
Flolan®	00173051900
Epoprostenol sodium	00703198501
Epoprostenol sodium	00703199501
Flolan®	20694011101
Flolan®	20694011201
Veletri®	66215040101
Veletri®	66215040201
Veletri®	66215040301
	**HCPCS**
Injection, epoprostenol, 0.5 mg	J1325
Iloprost	
	**NDC**
Ventavis®	10148010100
Ventavis®	10148010101
Ventavis®	10148010130
Ventavis®	10148010200
Ventavis®	10148010230
Ventavis®	12072010100
Ventavis®	12072010101
Ventavis®	12072010130
Ventavis®	12072010200
Ventavis®	12072010230
Ventavis®	66215030200
Ventavis®	66215030230
Ventavis®	66215030330
	**HCPCS**
Iloprost, inhalation solution, FDA-approved final product, non-compounded, administered through DME, unit dose form, 20 µg	Q4080

Table S1. Codes Used to Ascertain Pulmonary Hypertension Diagnoses, Drugs, Comorbidities, and Treatment-related Unwanted Events

**Description**	**Code**
Iloprost, inhalation solution, FDA-approved final product, non-compounded, administered through DME, unit dose form, up to 20 µg	Q4074
Treprostinil	**NDC**
Orenitram®	66302030001
Orenitram®	66302030010
Orenitram®	66302030201
Orenitram®	66302030210
Orenitram®	66302031001
Orenitram®	66302031010
Orenitram®	66302032501
Orenitram®	66302032510
Orenitram®	66302035001
Orenitram®	66302035010
Treprostinil injectable	00703066601
Treprostinil injectable	00703067601
Treprostinil injectable	00703068601
Treprostinil injectable	00703069601
Treprostinil injectable	00781342080
Treprostinil injectable	00781342580
Treprostinil injectable	00781342780
Treprostinil injectable	00781343080
Treprostinil injectable	42023020601
Treprostinil injectable	42023020701
Treprostinil injectable	42023020801
Treprostinil injectable	42023020901
Remodulin®	66302010101
Remodulin®	66302010201
Remodulin®	66302010501
Remodulin®	66302011001
Tyvaso® Starter Kit	66302020601
Tyvaso® Refill Kit	66302020602
Tyvaso®	66302020603
Tyvaso® Starter Kit (Institutional)	66302020604
	**HCPCS**
Injection, treprostinil, 1 mg	J3285
Injection, treprostinil sodium, 0.5 mg	S0114
Injection, treprostinil, 1 mg	Q4077
Treprostinil, inhalation solution, FDA-approved final product, non-compounded, administered through DME, unit dose form, 1.74 mg	J7686
Ambrisentan	**NDC**
Letairis®	61958080101
Letairis®	61958080102
Letairis®	61958080103
Letairis®	61958080105
Letairis®	61958080201
Letairis®	61958080202

Table S1. Codes Used to Ascertain Pulmonary Hypertension Diagnoses, Drugs, Comorbidities, and Treatment-related Unwanted Events

Description	Code
Letairis®	61958080203
Letairis®	61958080205
Bosentan	
Tracleer®	66215010103
Tracleer®	66215010106
Tracleer®	66215010203
Tracleer®	66215010206
Macitentan	
Opsumit®	66215050115
Opsumit®	66215050130
Riociguat	
Adempas®	50419025003
Adempas®	50419025291
Adempas®	50419025091
Adempas®	50419025203
Adempas®	50419025301
Adempas®	50419025303
Adempas®	50419025401
Adempas®	50419025491
Adempas®	50419025001
Adempas®	50419025191
Adempas®	50419025201
Adempas®	50419025403
Adempas®	50419025101
Adempas®	50419025103
Adempas®	50419025391
Sildenafil	
Revatio®	00069033621
Revatio®	00069033801
Revatio®	00069419068
Sildenafil citrate	00093551798
Sildenafil citrate	00378165777
Sildenafil citrate	00591378019
Sildenafil citrate	13668018505
Sildenafil citrate	13668018590
Sildenafil citrate	16714033801
Sildenafil citrate	31722077690
Sildenafil citrate	33342012110
Sildenafil citrate	42291073090
Sildenafil citrate	42291074990
Sildenafil citrate	42543000590
Sildenafil citrate	43063055010
Sildenafil citrate	43063055030
Sildenafil citrate	43063066830
Sildenafil citrate	43063067610
Sildenafil citrate	50268071715

Table S1. Codes Used to Ascertain Pulmonary Hypertension Diagnoses, Drugs, Comorbidities, and Treatment-related Unwanted Events

**Description**	**Code**
Sildenafil citrate	55111037290
Sildenafil citrate	55150016613
Sildenafil citrate	59762003301
Sildenafil citrate	60505340405
Sildenafil citrate	60505340409
Sildenafil citrate	65162035109
Sildenafil citrate	65862068890
Sildenafil citrate	68001017605
Sildenafil citrate	68084062221
Sildenafil citrate	68084086921
Tadalafil	
Adcirca®	66302046760
**Comorbidities**	
Digital ulcer	**ICD-9-CM**
Ulcer of other part of foot	707.15
Chronic ulcer of unspecified site	707.9
Chronic ulcer of other specified sites	707.8
	**ICD-10-CM**
Non-pressure chronic ulcer of lower limb, not elsewhere classified	L97
Other disorders of skin and subcutaneous tissue, not elsewhere classified	L98
Asthma	**ICD-9-CM**
Extrinsic asthma, unspecified	49300
Extrinsic asthma with status asthmaticus	49301
Extrinsic asthma with (acute) exacerbation	49302
Intrinsic asthma, unspecified	49310
Intrinsic asthma with status asthmaticus	49311
Intrinsic asthma with (acute) exacerbation	49312
Chronic obstructive asthma, unspecified	49320
Chronic obstructive asthma with status asthmaticus	49321
Chronic obstructive asthma with (acute) exacerbation	49322
Exercise induced bronchospasm	49381
Cough-variant asthma	49382
Asthma, unspecified type, unspecified	49390
Asthma, unspecified type, with status asthmaticus	49391
Asthma, unspecified type, with (acute) exacerbation	49392
	**ICD-10-CM**
Asthma	J45.x
Chronic obstructive pulmonary disease	**ICD-9-CM**
Bronchitis, not specified as acute or chronic	490
Chronic bronchitis	491.x
Emphysematous bleb	492.0
Other emphysema	492.8
Bronchiectasis without acute exacerbation	494.0
Bronchiectasis with acute exacerbation	494.1
Chronic airway obstruction, not elsewhere classified	496

Table S1. Codes Used to Ascertain Pulmonary Hypertension Diagnoses, Drugs, Comorbidities, and Treatment-related Unwanted Events

**Description**	**Code**
	**ICD-10-CM**
Bronchitis, not specified as acute or chronic	J40
Chronic bronchitis	J41.x
Unspecified chronic bronchitis	J42
Emphysema	J43.x
Chronic obstructive pulmonary disease	J44.x
Bronchiectasis	J47.x
Congenital heart diseases	**ICD-9-CM**
Common truncus	745.0
Transposition of great vessels	745.1x
Tetralogy of fallot	745.2
Common ventricle	745.3
Ventricular septal defect	745.4
Ostium secundum type atrial septal defect	745.5
Endocardial cushion defect	745.6x
Cor biloculare	745.7
Other bulbus cordis anomalies and anomalies of cardiac septal closure	745.8
Unspecified defect of septal closure	745.9
Congenital pulmonary valve anomaly	746.0x
Tricuspid atresia and stenosis, congenital	746.1
Ebstein's anomaly	746.2
Congenital stenosis of aortic valve	746.3
Congenital insufficiency of aortic valve	746.4
Congenital mitral stenosis	746.5
Congenital mitral insufficiency	746.6
Hypoplastic left heart syndrome	746.7
Other specified congenital anomalies of heart	746.8x
Unspecified congenital anomaly of heart	746.9
Anomaly of great veins	747.4x
	**ICD-10-CM**
Congenital malformations of cardiac chambers and connections	Q20.x
Congenital malformations of cardiac septa	Q21.x
Congenital malformations of pulmonary and tricuspid valves	Q22.x
Congenital malformations of aortic and mitral valves	Q23.x
Other congenital malformations of heart	Q24.x
Total anomalous pulmonary venous connection	Q26.2
Partial anomalous pulmonary venous connection	Q26.3
Congenital malformation of great vein, unspecified	Q26.9
Connective tissue diseases	**ICD-9-CM**
Lung involvement in systemic sclerosis	517.2
Diffuse diseases of connective tissue	710.x
Rheumatoid arthritis	714.0
Other rheumatoid arthritis with visceral or systemic involvement	714.2
Juvenile chronic polyarthritis	714.3x
Chronic postrheumatic arthropathy	714.4
	**ICD-10-CM**

Table S1. Codes Used to Ascertain Pulmonary Hypertension Diagnoses, Drugs, Comorbidities, and Treatment-related Unwanted Events

**Description**	**Code**
Systemic lupus erythematosus, organ or system involvement unspecified	M32.10
Systemic sclerosis	M34.x
Sicca syndrome, unspecified	M35.00
Sicca syndrome with keratoconjunctivitis	M35.01
Dermatopolymyositis, unspecified, organ involvement unspecified	M33.90
Polymyositis, organ involvement unspecified	M33.20
Other specified systemic involvement of connective tissue	M35.8
Multifocal fibrosclerosis	M35.5
Systemic involvement of connective tissue, unspecified	M35.9
Rheumatoid arthritis, unspecified	M06.9
Rheumatoid heart disease with rheumatoid arthritis of unspecified site	M05.30
Rheumatoid arthritis of unspecified site with involvement of other organs and systems	M05.60
Adult-onset Still's disease	M06.1
Unspecified juvenile rheumatoid arthritis of unspecified site	M08.00
Juvenile rheumatoid polyarthritis (seronegative)	M08.3
Pauciarticular juvenile rheumatoid arthritis, unspecified site	M08.40
Chronic postrheumatic arthropathy [Jaccoud], unspecified site	M12.00
Depression	**ICD-9-CM**
Major depressive affective disorder, single episode	296.2x
Major depressive affective disorder, recurrent episode	296.3x
Dysthymic disorder	300.4
Depressive disorder, not elsewhere classified	311
	**ICD-10-CM**
Major depressive disorder, single episode	F32.0–5, .9
Major depressive disorder, recurrent	F33.x
Dysthymic disorder	F34.1
Diabetes	**ICD-9-CM**
Secondary diabetes mellitus	249.x
Diabetes mellitus	250.x
Polyneuropathy in diabetes	357.2
Diabetic retinopathy	362.0x
Diabetic cataract	366.41
	**ICD-10-CM**
Diabetes mellitus due to underlying condition	E08.x
Drug or chemical induced diabetes mellitus	E09.x
Type 1 diabetes mellitus	E10.x
Type 2 diabetes mellitus	E11.x
Other specified diabetes mellitus	E13.x
Heart failure	**ICD-9-CM**
Rheumatic heart failure (congestive)	398.91
Hypertensive heart disease	402.x
Hypertensive heart and chronic kidney disease, malignant, with heart failure and with chronic kidney disease stage 1 through stage 4, or unspecified	404.01
Hypertensive heart and chronic kidney disease, malignant, with heart failure and with chronic kidney disease stage 5 or end stage renal disease	404.03

Table S1. Codes Used to Ascertain Pulmonary Hypertension Diagnoses, Drugs, Comorbidities, and Treatment-related Unwanted Events

Description	Code
Hypertensive heart and chronic kidney disease, benign, with heart failure and with chronic kidney disease stage 1 through stage 4, or unspecified	404.11
Hypertensive heart and chronic kidney disease, benign, with heart failure and chronic kidney disease stage 5 or end stage renal disease	404.13
Hypertensive heart and chronic kidney disease, unspecified, with heart failure and with chronic kidney disease stage 1 through stage 4, or unspecified	404.91
Hypertensive heart and chronic kidney disease, unspecified, with heart failure and chronic kidney disease stage 5 or end stage renal disease	404.93
Heart failure	428.x
	**ICD-10-CM**
Rheumatic heart failure	I09.81
Hypertensive heart disease with heart failure	I11.0
Hypertensive heart and chronic kidney disease with heart failure and stage 1 through stage 4 chronic kidney disease, or unspecified chronic kidney disease	I13.0
Hypertensive heart and chronic kidney disease with heart failure and with stage 5 chronic kidney disease, or end stage renal disease	I13.2
Heart failure, unspecified	I50.9
Left ventricular failure, unspecified	I50.1
Systolic (congestive) heart failure	I50.2x
Diastolic (congestive) heart failure	I50.3x
Combined systolic (congestive) and diastolic (congestive) heart failure	I50.4x
Hyperlipidemia	**ICD-9-CM**
Pure hypercholesterolemia	272.0
Pure hyperglyceridemia	272.1
Mixed hyperlipidemia	272.2
Hyperchylomicronemia	272.3
Other/unspecified hyperlipidemia	272.4
	**ICD-10-CM**
Pure hypercholesterolemia	E78.0
Pure hyperglyceridemia	E78.1
Mixed hyperlipidemia	E78.2
Hyperchylomicronemia	E78.3
Other hyperlipidemia	E78.4
Hyperlipidemia, unspecified	E78.5
Hypertension	**ICD-9-CM**
Hypertensive retinopathy	362.11
Essential hypertension	401.x
Hypertensive heart disease	402.x
Hypertensive chronic kidney disease	403.x
Hypertensive heart and chronic kidney disease	404.x
Secondary hypertension	405.x
Hypertensive encephalopathy	437.2
	**ICD-10-CM**
Hypertensive retinopathy	H35.03x
Essential (primary) hypertension	I10
Hypertensive heart disease	I11.x
Hypertensive chronic kidney disease	I12.x
Hypertensive heart and chronic kidney disease	I13.x

Table S1. Codes Used to Ascertain Pulmonary Hypertension Diagnoses, Drugs, Comorbidities, and Treatment-related Unwanted Events

**Description**	**Code**
Secondary hypertension	I15.x
Hypertensive encephalopathy	I67.4
Obesity	**ICD-9-CM**
Overweight and obesity	278.0x
BMI 30-39, adult	V85.3x
BMI ≥40, adult	V85.4x
	**ICD-10-CM**
Overweight and obesity	E66.x
BMI 30-39, adult	Z68.3x
BMI ≥40, adult	Z68.4x
Portal hypertension	**ICD-9-CM**
Portal hypertension	572.3
	**ICD-10-CM**
Portal hypertension	K76.6

Abbreviations: BMI, body mass index; COPD, chronic obstructive pulmonary disease; CPT-4, Current Procedural Terminology, 4th Edition; DME, durable medical equipment; FDA, Food and Drug Administration; HCPCS, Healthcare Common Procedure Coding System; ICD-9-CM, *International Classification of Diseases, Ninth Revision, Clinical Modification*; ICD-10-CM, *International Classification of Diseases, Tenth Revision, Clinical Modification*; NDC, National Drug Code; NKH-HC, nonketotic hyperglycemic-hyperosmolar coma.

Table S2. Prescribing Criteria for the Drugs of Interest

Drug	Route of Administration	FDA Label Indication	Reference
UPTRAVI® (selexipag)	Oral	UPTRAVI® is indicated for the treatment of pulmonary arterial hypertension (PAH, WHO Group I) to delay disease progression and reduce the risk of hospitalization for PAH.<br>Effectiveness was established in a long-term study in PAH patients with WHO Functional Class II-III symptoms.<br>Patients had idiopathic and heritable PAH (58%), PAH associated with connective tissue disease (29%), PAH associated with congenital heart disease with repaired shunts (10%).	FDA, Uptravi® Prescribing Information, https://www.accessdata.fda.gov/drugsatfda_docs/label/2017/207947s005lbl.pdf [accessed April 28, 2022]
Ventavis® (iloprost)	Inhalation	Ventavis® is a synthetic analog of prostacyclin indicated for the treatment of pulmonary arterial hypertension (PAH) (WHO Group 1) to improve a composite endpoint consisting of exercise tolerance, symptoms (NYHA Class), and lack of deterioration. Studies establishing effectiveness included predominately patients with NYHA Functional Class III-IV symptoms and etiologies of idiopathic or heritable PAH (65%) or PAH associated with connective tissue diseases (23%).	FDA, Ventavis® Prescribing Information, https://www.accessdata.fda.gov/drugsatfda_docs/label/2013/021779s014lbl.pdf [accessed April 28, 2022]
Remodulin® (treprostinil)	Continuous subcutaneous infusion (undiluted) is the preferred mode. Use intravenous (IV) infusion (dilution required) if subcutaneous infusion is not tolerated	Remodulin® is a prostacyclin vasodilator indicated for:<br>• Treatment of pulmonary arterial hypertension (PAH) (WHO Group 1) to diminish symptoms associated with exercise. Studies establishing effectiveness included patients with NYHA Functional Class II-IV symptoms and etiologies of idiopathic or heritable PAH (58%), PAH associated with congenital systemic-to-pulmonary shunts (23%), or PAH associated with connective tissue diseases (19%).<br>• Patients who require transition from Flolan®, to reduce the rate of clinical deterioration. The risks and benefits of each drug should be carefully considered prior to transition.	FDA, Remodulin® Prescribing Information, https://www.accessdata.fda.gov/drugsatfda_docs/label/2018/021272s026lbl.pdf [accessed April 28, 2022]

Abbreviations: FDA, US Food and Drug Administration; IV, intravenous; NYHA, New York Heart Association; PAH, pulmonary arterial hypertension; WHO, World Health Organization.
